# Occupational exposure to dusts and risk of renal cell carcinoma

**DOI:** 10.1038/bjc.2011.148

**Published:** 2011-05-03

**Authors:** S Karami, P Boffetta, P S Stewart, P Brennan, D Zaridze, V Matveev, V Janout, H Kollarova, V Bencko, M Navratilova, N Szeszenia-Dabrowska, D Mates, J Gromiec, A Slamova, W-H Chow, N Rothman, L E Moore

**Affiliations:** 1 Division of Cancer Epidemiology and Genetics, National Cancer Institute, NIH, DHHS, Occupational and Environmental Epidemiology Branch, 6120 Executive Boulevard, EPS 8121, Rockville, MD 20852, USA; 2 International Agency for Research on Cancer, Lyon 69008, France; 3 The Tisch Cancer Institute, Mount Sinai School of Medicine, New York, NY 10029, USA; 4 International Prevention Research Institute, Lyon 69006, France; 5 Stewart Exposure Assessments, LLC, Arlington, VA, USA (Formerly of the Division of Epidemiology and Genetics, National Cancer Institute, NIH, DHHS, Bethesda, MD 20852, USA); 6 Institute of Carcinogenesis, Cancer Research Centre, Moscow 115478, Russia; 7 Department of Preventive Medicine, Faculty of Medicine, Palacky University, Olomouc 77515, Czech Republic; 8 Institute of Hygiene and Epidemiology, First Faculty of Medicine Charles University, Prague 12800, Czech Republic; 9 Department of Cancer Epidemiology and Genetics, Masaryk Memorial Cancer Institute, Brno 65653, Czech Republic; 10 Department of Epidemiology, Nofer Institute of Occupational Medicine, Lodz 90950, Poland; 11 Institue of Public Health, Bucharest 76256, Romania; 12 Department of Chemical Hazards, Nofer Institute of Occupational Medicine, Lodz 91348, Poland

**Keywords:** renal cell carcinoma, occupational dust exposure, fibreglass, mineral wool, brick dust

## Abstract

**Background:**

Occupational exposures to dusts have generally been examined in relation to cancers of the respiratory system and have rarely been examined in relation to other cancers, such as renal cell carcinoma (RCC). Although previous epidemiological studies, though few, have shown certain dusts, such as asbestos, to increase renal cancer risk, the potential for other occupational dust exposures to cause kidney damage and/or cancer may exist. We investigated whether asbestos, as well as 20 other occupational dust exposures, were associated with RCC risk in a large European, multi-center, hospital-based renal case–control study.

**Methods:**

General occupational histories and job-specific questionnaires were reviewed by occupational hygienists for subject-specific information. Odds ratios (ORs) and 95% confidence intervals (95% CIs) between RCC risk and exposures were calculated using unconditional logistic regression.

**Results:**

Among participants ever exposed to dusts, significant associations were observed for glass fibres (OR: 2.1; 95% CI: 1.1–3.9), mineral wool fibres (OR: 2.5; 95% CI: 1.2–5.1), and brick dust (OR: 1.5; 95% CI: 1.0–2.4). Significant trends were also observed with exposure duration and cumulative exposure. No association between RCC risk and asbestos exposure was observed.

**Conclusion:**

Results suggest that increased RCC risk may be associated with occupational exposure to specific types of dusts. Additional studies are needed to replicate and extend findings.

Occupational dust exposures are generally thought to adversely affect the respiratory system and in some studies have been associated with cancers of the lung, trachea, bronchus, oral cavity, pharynx, and larynx (Maier *et al*, 1990; Marsh *et al*, 1990; Alberg *et al*, 2007). Although the kidney is not in direct contact with dusts through inhalation, increased risk of kidney damage and cancer have been observed in occupational studies of asbestos and crystalline silica exposures (Kolev *et al*, 1970; Markovic and Arambasic, 1971; Enterline *et al*, 1987; Smith *et al*, 1989; Pesch *et al*, 2000; El-Safty *et al*, 2003; Steenland, 2005; Lipworth *et al*, 2006; Pascual and Borque, 2008; Roggli *et al*, 2004, pp 224–225), particulates that are primarily inhaled in occupational settings and mainly linked to respiratory cancers (Alberg *et al*, 2007).

Because kidney cancer and damage has previously been associated with occupational dust exposures (Kolev *et al*, 1970; Markovic and Arambasic, 1971; Enterline *et al*, 1987; El-Safty *et al*, 2003; Steenland, 2005; Pascual and Borque, 2008), we examined this association in the Central and Eastern European Renal Cell Carcinoma (CEERCC) study, where we also were able to extend these findings to dusts other than those studied previously. Here, we investigated whether occupational dust exposures were associated with an increased risk of renal cell carcinoma (RCC) among participants enrolled in a large, multi-centered renal case–control study conducted in Central and Eastern Europe, an area with historically heavy industrial exposures and one of the highest rates of RCC in the world (International Agency for Cancer Research, GLOBACAN, 2011).

## Materials and methods

The CEERCC study is a large hospital-based multi-center case–control study of renal cancer conducted across four Central and Eastern European countries (Moscow, Russia; Bucharest, Romania; Lodz, Poland; and Prague, Olomouc, Ceske-Budejovice, and Brno, Czech Republic) between 1999 and 2003. Details of this study have been previously reported (van der Hel *et al*, 2008; Heck *et al*, 2010). Newly diagnosed renal cancer patients, greater than 20 years of age were recruited for participation. Controls admitted to the same hospital as cases, diagnosed with conditions unrelated to smoking or urological disorders (with the exception of benign prostatic hyperplasia) were frequency matched to cases on age (±3 years), sex, and place of residence. No single disease made up greater than 20% of the control group. Some controls also were recruited in parallel for studies of lung and head and neck cancers (Scelo *et al*, 2004; Hashibe *et al*, 2007). All eligible participants were required to have lived within the study areas for at least 1 year before enrolment. Response rates for participation across study centres ranged from 90 to 99% for cases and from 90 to 96% for controls. Overall, 1097 histologically confirmed RCC (IDC-O-2 code 64) cases and 1476 controls were included in the study. All institutional review boards of all participating centres and organisations approved the study and all subjects provided written informed consent.

During hospitalisation and within three months of diagnosis for cases, all participants were interviewed in-person by trained interviewers blinded to case–control status. Details regarding the questionnaires have been previously reported (Heck *et al*, 2010). First, a standardised questionnaire was administered to collect information on demographic characteristics, medical histories, and lifestyle factors. Subsequently, lifetime occupational information for all jobs held for ⩾12 months duration was ascertained through the use of a general occupational questionnaire. Information included job title, detailed tasks, type of employer, and year of beginning and ending employment. Data regarding broad categories of exposure (e.g., dusts, pesticides, liquids, etc.) was also collected. Where employment in specific jobs or industries was likely to entail exposure to known or suspected occupational carcinogens of interest, job-specific questionnaires were administered and information regarding sources of exposure was identified. Subjects who were occupationally exposed to dust, for example, were asked to classify their exposure as sand, cement, concrete, metal, wood, other (‘please specify’), and so on.

Chemists, industrial hygienists, and occupational physicians from each centre, who were trained by the study's lead industrial hygienist, reviewed all occupational questionnaires. The exposure assessors rated the frequency and intensity of occupational exposure to 72 specific agents of interest based on historical data from the general and job-specific occupational questionnaires as well as the experts’ knowledge. For the purposes of this manuscript, all agents associated with occupational exposure to dusts were examined. Frequency of occupational exposure from various types of dust was assessed as <5% (<2 h), 5%–30% (2–12 h), and >30% (>12 h) of total working time in a 40-h workweek. Intensity of exposure was assessed as low, medium, and high, based on agent-specific categories anchored to measurement data and jobs. For each agent assessed, the experts also noted the degree of confidence of their assessment, categorised as possible (<40%), probable (40–90%), or definite (>90%). As the degree of confidence for the vast majority (>91%) of agents in this study were assessed as probable or definite, analyses were restricted to exposures with a high (⩾40%) confidence of exposure. Experts were blinded to the case–control status of participants while reviewing occupational histories and assessing exposures to specific agents.

The reliability of the experts’ assessments for all agents across the seven study centres was evaluated with an inter-team agreement study of 19 job descriptions and 54 exposures (Durusoy *et al*, 2006) and found that the overall quality was comparable among expert teams. Specifically, through the use of *κ* scores, agreement for the presence or absence of an exposure among teams was excellent (*κ*>0.75) whereas agreement with regards to confidence, intensity, and frequency of exposures was fair to good (*κ* between 0.4–0.75) (Mannetje *et al*, 2003).

For each subject, exposure metrics for each dust agent that were assessed included the following: (1) ever exposure, (2) duration of exposure expressed as the total number of years subjects worked in a job in which exposure was possible, and (3) cumulative exposure, calculated as the duration of exposure in years for each job multiplied by the midpoint of the frequency category and by the intensity weight of the job, summed across all of the subject's jobs. To calculate cumulative exposure across jobs with varying frequencies of exposure, frequency weights (0.03, 0.175, and 0.65, respectively) were assigned to the three frequency categories, corresponding to the midpoint of the ranges.

To estimate RCC risk and associations with occupational exposure history, odds ratios (ORs) and 95% confidence intervals (95% CIs) were calculated using unconditional logistic regression models adjusting for sex, age (continuous), center, smoking status (never, ever), body mass index (BMI) at interview, and self-reported hypertension (no, yes). Occupational co-exposures with a significant (*P*<0.05) *r*^2^>0.50, identified using Spearman correlation coefficients, that were shown to modify OR and 95% CI values by at least 10% were also included for adjustment. The strength of associations between co-exposures is presented as a matrix in Supplementary Table 1. The number of women exposed in our study was too small to warrant separate analyses, so both sexes were combined. Subgroup analyses with a 10-year and a 20-year lag period between exposure and diagnosis were conducted to restrict analyses to subjects with a sufficient latency period from occupational exposure to cancer diagnosis. The results of the 10-year lag are not presented because findings are similar to that of the 20-year lag. Subjects were evaluated as never and ever exposed groups. Duration and cumulative exposure categories were divided into level of exposure based on the 50th percentile cut-point among all subjects. All analyses were conducted in STATA 9.0 (STATA Corporation, College Station, TX, USA).

## Results

Selected characteristics of the study population by case–control status are shown in Table 1. Participants were similar with respect to age. Cases, however, were more likely to be female and to have excess BMI and hypertension. Cases were less likely to smoke, yet after adjusting for sex, age, study center, BMI, and self-reported hypertension the inverse association with smoking, as previously reported, was no longer significant (van der Hel *et al*, 2008).

Renal cancer risk associations by occupational dust exposure are provided in Table 2. Increased ORs were observed among subjects ever occupationally exposed to glass fibres (OR: 2.1; 95% CI: 1.1–3.9), mineral wool fibres (OR: 2.5; 95% CI: 1.2–5.1), and brick dust (OR: 1.5; 95% CI: 1.0–2.4). Results by duration and cumulative exposure are shown in Table 3 for agents for which a significant association was found in the analysis of ever exposure, duration of exposure, or cumulative exposure. Duration of exposure in years revealed a two- to three-fold increase in cancer risk for occupational exposures to glass fibres (*P*-trend=0.03), mineral wool fibres (*P*-trend=0.02), and brick dust (*P*-trend=0.01). Similar findings were also shown for cumulative exposure to these dust agents. RCC risk was lower among individuals exposed to graphite dust by duration in years (*P*-trend=0.02) and cumulative exposure (*P*-trend=0.049), though ever exposure to graphite dust was not associated with risk. Exposure to asbestos and other dust agents listed in Table 2 were not associated with RCC risk by duration or cumulative exposure (data not shown).

The relationship between renal cancer risk and these occupational dust exposures was also examined allowing for a 20-year lag between exposure and diagnosis (Supplementary Table 2). Similar associations between RCC risk and exposure were observed; however, only moderate associations with risk were observed for occupational exposure to glass fibre and graphite dust, likely due to lower number of exposed subjects.

## Discussion

RCC risk was associated with occupational exposure to specific types of dusts, specifically glass fibre, mineral wool fibre, and brick dust. Exposure by duration and cumulative exposure estimates showed similar associations. In contrast, an inverse association was found for graphite dust exposure, when analysed by duration and cumulative exposure.

Positive associations between occupational exposures to glass fibres, mineral wool fibres, and/or brick dust have been reported for cancers of the respiratory system (Olsen and Jensen, 1984; Siemiatycki *et al*, 1986; Neuberger *et al*, 1988; Marsh *et al*, 1990; Shannon *et al*, 2005). Results for renal cancer risk, however, have been inconsistent (Olsen and Jensen, 1984; Siemiatycki *et al*, 1986; Marsh *et al*, 1990; Burnett and Dosemeci, 1994; Mellemgaard *et al*, 1994; Robinson *et al*, 1995; Stone *et al*, 2004; Shannon *et al*, 2005). For example, increased kidney cancer risk was reported in a large Canadian cohort of 2557 male glass fibre-manufacturing workers (Shannon *et al*, 2005); yet, no association between kidney cancer risk and exposure to glass fibres was reported among 4008 female workers in 10 United States fibreglass-manufacturing plants (Stone *et al*, 2004). Similarly, in a cohort of United States man-made mineral fibre workers, a lack of association between kidney cancer mortality risk and exposure to airborne fibre concentrations among mineral wool and fibreglass plant workers was reported by Marsh *et al* (1990). No relationship between kidney cancer risk and exposure to wool or mineral wool was observed in a Montreal multi-cancer case–control study of 100 kidney cancer patients (Siemiatycki *et al*, 1986) or in a cohort of mineral wool production workers in Denmark (Olsen and Jensen, 1984). The lack of supporting evidence from cohort studies, therefore, reduces the plausibility of an association between RCC risk and exposure to both glass and mineral wool fibres. Among workers suspected of brick dust exposure, a nonsignificant elevation in RCC risk was observed among bricklayers in a small population-based case–control study in Europe (Mellemgaard *et al*, 1994) and elevated kidney cancer mortality risk was seen among concrete/terrazzo finishers in a surveillance study of construction workers across the United States (Robinson *et al*, 1995). On the other hand, using occupational mortality surveillance data, the National Institute for Occupational Safety and Health reported a significant increase in kidney cancer risk among female workers employed in the pottery industry, where silica exposure occurs (Burnett and Dosemeci, 1994). Possible explanations for the lack of consistent findings across studies include insufficient power due to limited number of cases or exposed subjects, exposure misclassification, or co-existing occupational/environmental exposures that were not accounted for; therefore, additional sufficiently powered and well-conducted studies are needed to determine if these associations are real.

Although it is questionable whether inhaled dust particles can reach the kidney, the relationship between renal cancer and the dust exposures observed in our study is plausible as they contain silica; however, the relationship is less plausible for the fibres as the silica is bound. In 1996, the International Agency for Cancer Research classified crystalline silica as a group 1 human carcinogen based on epidemiological and laboratory animal studies that demonstrated silica exposure was associated with increased lung cancer (IARC, 1997). Furthermore, growing scientific evidence over the past few decades suggests that chronic silica exposure can induce nephrotoxicity and cause fibrosis, glomerulonephritis, and degenerative changes in tubular epithelium (Kolev *et al*, 1970; Markovic and Arambasic, 1971; IARC, 1997; El-Safty *et al*, 2003; Steenland, 2005). In a recently published review of occupational epidemiological studies, Steenland (2005) reported excess risk of end stage renal disease among silica-exposed workers . Excess kidney cancer risk has also been reported among silica-exposed workers in two earlier cohort studies (Cooper *et al*, 1992; Hobbesland *et al*, 1999). In our case–control study, however, no association between occupational respirable free crystalline silica exposure and RCC risk was observed. Animal studies have demonstrated that silica exposure may affect DNA replication, gene expression, and repair (Ding *et al*, 2002; Fubini and Hubbard, 2003). Animal studies have also shown silica to interfere with mitotic spindle formation and the segregation of chromosomes, which could eventually induce aneuploidy (Ding *et al*, 2002; Fubini and Hubbard, 2003; Gao *et al*, 2009). The only cytogenetic study on crystalline silica exposure reported an increased prevalence of sister chromatid exchanges and chromosomal aberrations in peripheral blood lymphocytes (Nagalakshmi *et al*, 1995). In prospective cohort studies, chromosomal aberrations have been associated with future cancer risk (Bonassi *et al*, 2000; Hagmar *et al*, 2004; Vodicka *et al*, 2010). Because the exact mechanism involved in the carcinogenicity of silica remains unclear and it is questionable whether silica from fibres is available for bioactivity, until additional epidemiological studies are conducted, the possible association between renal carcinoma and silica exposure remains uncertain.

In our study a large proportion of subjects who were occupationally exposed to glass (57% of cases and 42% of controls) or mineral wool (86% of cases and 64% of controls) fibres were also occupationally exposed to asbestos. The relationship between RCC risk and glass and mineral wool fibres in our study may also be explained by the asbestos-like properties shared by these fibres (Kamp, 2009; Agency for Toxic Substances and Diseases Registry, http://www.atsdr.cdc.gov/toxprofiles/tp61.pdf and http://www.atsdr.cdc.gov/toxprofiles/tp161.pdf). Similar to asbestos, inhaled mineral wool and glass fibre particulates are capable of depositing deep within the lungs, due to their needle-like dimensions, reaching the alveoli, and provoking macrophages to attack (Agency for Toxic Substances and Diseases Registry, http://www.atsdr.cdc.gov/toxprofiles/tp61.pdf and http://www.atsdr.cdc.gov/toxprofiles/tp161.pdf). The ingested particles produce an inflammatory response where fibroblasts deposit, produce, and proliferate tissue leading to the development of cancer (Agency for Toxic Substances and Diseases Registry, http://www.atsdr.cdc.gov/toxprofiles/tp61.pdf and http://www.atsdr.cdc.gov/toxprofiles/tp161.pdf). Evidence of asbestos inhalation associated with kidney cancer in studies of workers has been inconsistent (Partanen *et al*, 1991; Sali and Boffetta, 2000; Agency for Toxic Substances and Diseases Registry, http://www.atsdr.cdc.gov/toxprofiles/tp61.pdf and http://www.atsdr.cdc.gov/toxprofiles/tp161.pdf). In our study, no association between RCC risk and occupational exposure to asbestos was shown. Moreover, a meta-analysis of 37 cohort studies conducted on workers suspected of asbestos exposure revealed limited evidence of an association between kidney cancer risk and asbestos exposure. Standardised mortality ratios/standardised incidence ratios (SMR/SIR) across the studies ranged from 0.22 to 5.00 and pooled analyses revealed no significant findings for chrysotile or amphibole asbestos exposures (Sali and Boffetta, 2000). Thus, it is unclear whether our findings of an association with glass and mineral wool fibres are real.

The inverse association between graphite dust exposure and RCC risk in our study was unexpected. Results are suspected to be chance related, due to the small number of exposed participants (cases *N*=12, controls *N*=32) and limited power to conclude statistically meaningful result for ever exposure (47%) or for trend analysis for cumulative exposure (58%). Furthermore, the only study to examine cancer risk in graphite electric workers reported a nonsignificant 1.8-fold increased SMR for kidney cancer (Merlo *et al*, 2004).

High participation rates, a large sample size, inclusion of only newly diagnosed and histologically confirmed cancers, use of job-specific questionnaire modules to collect individual-specific exposure information, and expert-based exposure assessment teams were some of the strengths of our study. Sufficient statistical power to detect relatively small associations between RCC risk and exposure (ever *vs* never) to occupational dust was possible due to the large sample size of the study; however, power for some of the exposure–response relationships was limited due to the small number of exposed subjects. Other limitations of our study included the possibility of inaccurate or incomplete recall of all occupational histories and the use of hospital-based controls, which may not be representative of the general non-diseased reference population, even though we attempted to address this issue by recruiting controls with a wide range of disease diagnoses. Moreover, lack of data regarding personal protective equipment, ventilation at specific jobs, working conditions, and possible environmental exposures to dust (i.e., pollution), may have increased the likelihood of exposure misclassification and possibly confounded results. Although exposure misclassification is always of concern, the result of any misclassification would likely diminish the elevated risks and significant trends towards the null if the missclassification were non-differential. In our study, the prevalence of occupational exposures to certain dusts (e.g. asbestos silica, chrysotile asbestos, and amphibole asbestos) were compatible to the prevalence of exposure reported in other recently published case–control studies in Central and Eastern Europe (Krstev *et al*, 2005; Zeka *et al*, 2006; Carel *et al*, 2007), which strengthens our exposure assessment confidence. Even so, aassessment of jobs and exposures obtained through interview should be critically evaluated, as the likelihood of exposure misclassification is higher than for studies with actual exposure measurements. For this reason, analysis of dust exposures was evaluated among jobs with only high confidence exposures, which were assessed by raters blinded to disease status. Restricting the analyses to these subjects generally increased the risks slightly. Additionally, although we were able to control for known RCC risk factors, such as self-reported hypertension, smoking, and BMI, other potential risk modifiers (i.e., other occupational exposures, genetics, diet, environmental exposures, working conditions) were not considered and may have biased our results due to uncontrolled confounding. Self-reported hypertension status was unconfirmed and was a potential source of misclassification. Finally, 63 tests were preformed of which 11 were positively associated with RCC risk; therefore, the possibility of chance findings due to multiple comparison tests is probable. However for some agents, such as brick dust, this is unlikely as the association with RCC risk became stronger with increasing years and cumulative exposure and restriction of jobs with high confidence.

In summary, the results of our study found a possible association between RCC risk and workers in Central and Eastern Europe exposed to glass fibre, mineral wool fibre, and brick dust. When analyses were restricted to high-confidence exposures, the association between these dust agents and RCC risk became stronger and statistically significant. Additionally, exposure–response relationships for these agents showed both a significant and linear increase in RCC risk by cumulative exposure and duration (except for glass fibres) of exposure. Similar associations were also observed when analyses were examined allowing for a 20-year lag between exposure and diagnosis. The lack of association between RCC and occupational exposures to silica and asbestos in our study, however, justifies the need for further investigation. Our observed associations also require replication before meaningful inferences can be concluded.

## Figures and Tables

**Table 1:** General characteristics of participants in the CEERCC Study

	**Case**		**Control**	
**Variables**	***N***	**%^a^**	***N***	**%^a^**
Participants	1097	42.6	1476	57.4
				
*Sex*				
Males	648	59.1	952	64.5
Females	449	40.9	524	35.5
				
*Age at interview (in years)*				
<45	86	7.8	122	11.1
45–54	278	25.3	379	34.5
55–64	335	30.5	460	41.9
65–74	353	32.2	452	41.2
75+	45	4.1	63	5.7
				
Mean age (s.d.)	59.6 years (10.3)		59.3 years (10.3)	
		
*Centre*				
Bucharest, Romania	95	8.7	160	10.8
Lodz, Poland	99	8.7	198	13.4
Moscow, Russia	317	28.9	463	31.4
Czech Republic^b^	586	53.4	655	44.4
				
*BMI at interview (kg m* ^*−2*^ *)*				
<25	327	29.8	532	36.0
25–29.9	476	43.4	620	42.0
30+	294	26.8	324	22.0
				
*Tobacco status*				
Never	510	46.5	599	40.6
Ever	584	53.2	874	59.2
				
*Self-reported hypertension*				
No	600	54.7	906	61.4
Yes	496	45.2	569	38.6

Abbreviations: BMI=body mass index; CEERCC=Central and Eastern European Renal Cell Carcinoma.

a Due to missing values, some categories do not sum to 100%.

b Brno, Olomouc, Prague, Ceske-Budejovice.

**Table 2:** Ever occupationally exposed to dusts and risk of renal cell carcinoma

	**High confidence exposures**					
	**Case**		**Control**			
	***N***	**%**	***N***	**%**	**OR**	**95% CI**
*Inorganic insulation dust* a						
Unexposed	750	91.9	1100	93.5	1.0	
Exposed	66	8.1	77	6.5	1.3	0.9–2.1
						
*Asbestos* b						
Unexposed	738	90.3	1062	90.5	1.0	
Exposed	79	9.7	112	9.5	0.8	0.5–1.2
						
*Chrysotile asbestos* c						
Unexposed	778	94.8	1107	93.7	1.0	
Exposed	43	5.2	75	6.3	0.7	0.4–1.2
						
*Amphibole asbestos* d						
Unexposed	800	97.1	1151	97.3	1.0	
Exposed	24	2.9	32	2.7	1.4	0.6–3.1
						
*Glass fibres* a						
Unexposed	797	96.6	1165	98.4	1.0	
Exposed	28	3.4	19	1.6	2.1	1.1–3.9
						
*Mineral wool fibres* a						
Unexposed	803	97.3	1167	98.8	1.0	
Exposed	22	2.7	14	1.2	2.5	1.2–5.1
						
*Abrasive dust*						
Unexposed	669	81.6	947	80.1	1.0	
Exposed	151	18.4	236	19.9	0.9	0.7–1.2
						
*Sand*						
Unexposed	657	79.6	881	74.8	1.0	
Exposed	168	20.4	297	25.2	0.9	0.7–1.1
						
*Respirable free crystalline silica*						
Unexposed	774	93.9	1117	94.3	1.0	
Exposed	50	6.1	67	5.7	1.0	0.7–1.5
						
*Concrete dust* e						
Unexposed	726	87.9	1028	87.0	1.0	
Exposed	100	12.1	154	13.0	0.8	0.6–1.1
						
*Cement dust* f ^,^ g						
Unexposed	718	86.9	1020	86.1	1.0	
Exposed	108	13.1	164	13.9	1.3	0.8–2.0
						
*Brick dust* f						
Unexposed	753	91.3	1103	93.2	1.0	
Exposed	72	8.7	80	6.8	1.5	1.0–2.4
						
*Coal dust*						
Unexposed	783	94.8	1121	94.5	1.0	
Exposed	43	5.2	65	5.5	0.9	0.6–1.4
						
*Carbon black*						
Unexposed	819	99.3	1167	98.5	1.0	
Exposed	6	0.7	18	1.5	0.5	0.2–1.2
						
*Soot*						
Unexposed	784	95.1	1119	94.7	1.0	
Exposed	40	4.9	63	5.3	0.9	0.6–1.3
						
*Coke dust* h						
Unexposed	814	98.5	1164	98.1	1.0	
Exposed	12	1.5	22	1.9	0.7	0.3–1.6
						
*Graphite dust*						
Unexposed	813	98.5	1153	97.3	1.0	
Exposed	12	1.5	32	2.7	0.5	0.3–1.0
						
*Wood dust*						
Unexposed	742	89.9	1082	91.5	1.0	
Exposed	83	10.1	101	8.5	1.2	0.9–1.7
						
*Hard wood dust* i						
Unexposed	798	96.6	1141	96.4	1.0	
Exposed	28	3.4	43	3.6	0.7	0.4–1.3
						
*Soft wood dust* j						
Unexposed	760	92.0	1095	92.4	1.0	
Exposed	66	8.0	90	7.6	1.3	0.8–2.0
						
*Ash*						
Unexposed	808	97.8	1152	97.2	1.0	
Exposed	18	2.2	33	2.8	0.6	0.4–1.2

Abbreviations: BMI=body mass index; CI=confidence interval; OR=odds ratio.

Adjusted for age, sex, centre, BMI, self-reported hypertension, and smoking status (ever, never).

High confidence exposures include only those exposures assessed with a confidence of probable (40–90%) or definite (>90%).

Occupational exposures to ceramic fibres (cases *N*=1; controls *N*=1) and charcoal dust (cases *N*=1; controls *N*=1) are not shown due to small number of exposed.

Model also adjusted for occupational:

a Asbestos exposure.

b Inorganic insulation dust exposure.

c Amphibole asbestos exposure.

d Chrysotile asbestos exposure.

e Brick dust exposure.

f Concrete dust exposure.

g Sand exposure.

h Coke combustion fume exposure.

i Hard wood dust exposure.

j Soft wood dust exposure.

**Table 3:** Occupational dust exposures and risk of renal cell carcinoma

**Duration of Exposure**							**Cumulative Exposure**						
	**Case**	**Control**						**Case**	**Control**				
	***N***	***N***	**OR**	**95% CI**		***P*-trend**		***N***	***N***	**OR**	**95% CI**		***P*-trend**
*Glass fibres* a													
Unexposed	797	1165	1.0				Unexposed	797	1165	1.0			
⩽12.00	15	10	2.2	0.9	5.0		⩽0.05	12	10	1.6	0.7	3.8	
>12.00	13	9	2.0	0.8	4.8		>0.05	16	9	2.6	1.1	6.2	
						0.03							0.02
*Mineral wool fibres* a													
Unexposed	803	1167	1.0				Unexposed	803	1167	1.0			
⩽12.00	11	8	2.2	0.8	5.6		⩽0.06	9	7	2.0	0.7	5.7	
>12.00	11	6	2.9	1.0	8.1		>0.06	13	7	2.9	1.1	7.6	
						0.02							0.02
*Brick dust* b													
Unexposed	753	1103	1.0				Unexposed	753	1103	1.0			
⩽11.00	31	48	1.1	0.7	2.0		⩽1.40	36	42	1.4	0.8	2.5	
>11.00	41	32	2.1	1.2	3.6		>1.40	36	38	1.7	0.9	2.9	
						0.01							0.049
*Graphite dust*													
Unexposed	813	1153	1.0				Unexposed	813	1153	1.0			
⩽11.00	10	12	1.2	0.5	2.8		⩽3.08	7	15	0.7	0.3	1.8	
>11.00	2	20	0.1	0.0	0.6		>3.08	5	17	0.4	0.1	1.1	
						0.02							0.049

Abbreviations: BMI=body mass index; CI=confidence interval; OR=odds ratio.

Adjusted for age, sex, centre, BMI, self-reported hypertension, and smoking status (ever, never).

Includes only high confidence exposures, those exposures assessed with a confidence of probable (40–90%) or definite (>90%).

Model also adjusted for occupational:

a Asbestos exposure.

b Concrete dust exposure.
