# Supplementary material for: Occupational exposure to dusts and risk of renal cell carcinoma
Source: Br J Cancer. 2011 May 3;104(11):1797–803. doi: 10.1038/bjc.2011.148 (PMC3111161; doi:10.1038/bjc.2011.148)
Supplement: Supplementary Tables [file bjc2011148x1.doc]

| **Supplemental Table 1. Spearman correlation between dust exposures** | | | | | | | | | | | | | | | | | | | | | |
| --- | --- | --- | --- | --- | --- | --- | --- | --- | --- | --- | --- | --- | --- | --- | --- | --- | --- | --- | --- | --- | --- |
| Dust Exposures | Inorganic Insulation Dust | Asbestos | Chrysotile Asbestos | Amphibole Asbestos | Glass Fibers | Mineral Wool Fibers | Abrasive Dust | Sand | Respirable Free Crystalline Silica | Concrete Dust | Cement Dust | Brick Dust | Coal Dust | Carbon Black | Soot | Coke Dust | Graphite Dust | Wood Dust | Hard Wood Dust | Soft Wood Dust | Ash |
| Inorganic Insulation Dust | 1.000 |  |  |  |  |  |  |  |  |  |  |  |  |  |  |  |  |  |  |  |  |
| Asbestos | 0.588* | 1.000 |  |  |  |  |  |  |  |  |  |  |  |  |  |  |  |  |  |  |  |
| Chrysotile Asbestos | 0.348* | 0.744* | 1.000 |  |  |  |  |  |  |  |  |  |  |  |  |  |  |  |  |  |  |
| Amphibole Asbestos | 0.314* | 0.527* | 0.694* | 1.000 |  |  |  |  |  |  |  |  |  |  |  |  |  |  |  |  |  |
| Glass Fibers | 0.434* | 0.198* | 0.225* | 0.295* | 1.000 |  |  |  |  |  |  |  |  |  |  |  |  |  |  |  |  |
| Mineral Wool Fibers | 0.444* | 0.319* | 0.242* | 0.284* | 0.421* | 1.000 |  |  |  |  |  |  |  |  |  |  |  |  |  |  |  |
| Abrasive Dust | 0.229* | 0.285* | 0.208* | 0.159* | 0.073† | 0.087* | 1.000 |  |  |  |  |  |  |  |  |  |  |  |  |  |  |
| Sand | 0.175* | 0.176* | 0.174* | 0.176* | 0.141* | 0.136* | 0.106* | 1.000 |  |  |  |  |  |  |  |  |  |  |  |  |  |
| Respirable Free Crystalline Silica | 0.070† | 0.165* | 0.149* | 0.223* | 0.077* | 0.076* | 0.120* | 0.294* | 1.000 |  |  |  |  |  |  |  |  |  |  |  |  |
| Concrete Dust | 0.262* | 0.153* | 0.128* | 0.124* | 0.183* | 0.192* | 0.075* | 0.487* | 0.060† | 1.000 |  |  |  |  |  |  |  |  |  |  |  |
| Cement Dust | 0.199* | 0.125* | 0.098* | 0.117* | 0.175* | 0.147* | 0.029 | 0.602* | 0.066† | 0.723* | 1.000 |  |  |  |  |  |  |  |  |  |  |
| Brick Dust | 0.311* | 0.226* | 0.199* | 0.152* | 0.188* | 0.252* | 0.042 | 0.382* | 0.045† | 0.595* | 0.560* | 1.000 |  |  |  |  |  |  |  |  |  |
| Coal Dust | 0.062† | 0.089* | 0.062† | 0.086* | 0.014 | 0.059† | 0.047† | 0.159* | 0.314* | 0.035 | 0.048† | 0.055† | 1.000 |  |  |  |  |  |  |  |  |
| Carbon Black | 0.050† | 0.221* | 0.233* | 0.344* | 0.019 | -0.014 | 0.074† | 0.139 | 0.291* | 0.005 | 0.017 | 0.008 | 0.187* | 1.000 |  |  |  |  |  |  |  |
| Soot | 0.090* | 0.198* | 0.163* | 0.209* | 0.017 | 0.064† | 0.077* | 0.127* | 0.209* | 0.030 | 0.037 | 0.073† | 0.398* | 0.398* | 1.000 |  |  |  |  |  |  |
| Coke Dust | 0.065† | 0.189* | 0.115* | 0.157* | 0.040 | 0.049† | 0.066† | 0.087* | 0.219* | -0.010 | 0.037 | -0.004 | 0.257* | 0.336* | 0.421* | 1.000 |  |  |  |  |  |
| Graphite Dust | 0.033 | 0.166* | 0.139* | 0.198* | 0.005 | -0.019 | 0.096* | 0.142* | 0.307* | -0.033 | -0.014 | -0.013 | 0.121* | 0.458* | 0.261* | 0.296* | 1.000 |  |  |  |  |
| Wood Dust | 0.108* | 0.049† | 0.018 | 0.013 | 0.069† | 0.045 | 0.136* | 0.145* | 0.123* | 0.171* | 0.174* | 0.160* | 0.102* | 0.050† | 0.081* | 0.045 | 0.052† | 1.000 |  |  |  |
| Hard Wood Dust | 0.059† | 0.055† | 0.015 | 0.019 | 0.012 | 0.041 | 0.135* | 0.040 | 0.049† | 0.047† | 0.041 | 0.032 | 0.086* | 0.058† | 0.056† | -0.003 | 0.029 | 0.607* | 1.000 |  |  |
| Soft Wood Dust | 0.093* | 0.043 | 0.016 | 0.012 | 0.041 | 0.040 | 0.120* | 0.150* | 0.097* | 0.153* | 0.159* | 0.159* | 0.082* | 0.060† | 0.065† | 0.025 | 0.051† | 0.901* | 0.617* | 1.000 |  |
| Ash | 0.117* | 0.193* | 0.167* | 0.158* | 0.048† | 0.059† | 0.103* | 0.056† | 0.148* | 0.002 | 0.028 | 0.059† | 0.398* | 0.265* | 0.539* | 0.456* | 0.184* | 0.064† | 0.058† | 0.053† | 1.000 |
| † P-value<0.05; *P-value<0.001 | | | | | | | | | | | | | | | | | | | | | |

| **Supplemental Table 2 Occupational dust exposures and risk of renal cell carcinoma with a 20 year lag period** | | | | | | | | | | | | | | | | | | | | |
| --- | --- | --- | --- | --- | --- | --- | --- | --- | --- | --- | --- | --- | --- | --- | --- | --- | --- | --- | --- | --- |
| **Ever Exposed** | | | | | |  | **Duration of Exposure** | | | | | |  | **Cumulative Exposure** | | | | | | |
|  | Case | Control |  | | |  |  | Case | Control |  | | |  |  | Case | Control |  | | | |
|  | N | N | OR | 95% CI | p-trend | |  | N | N | OR | 95% CI | p-trend | |  | N | N | OR | 95% CI | | p-trend |
| **Glass Fibers ^a^** | | | | | | | | | | | | | | | | | | | | |
| Unexposed | 797 | 1165 | 1.0 |  |  |  | Unexposed | 797 | 1165 | 1.0 |  |  |  | Unexposed | 797 | 1165 | 1.0 |  | |  |
| Exposed | 23 | 19 | 1.8 | 0.9-3.3 | 0.09 |  | < 12.00 | 10 | 10 | 1.5 | 0.6-3.8 |  |  | < 0.05 | 9 | 10 | 1.2 | 0.5-3.2 | |  |
|  |  |  |  |  |  |  | > 12.00 | 13 | 9 | 2.0 | 0.8-4.9 |  |  | > 0.05 | 14 | 9 | 2.4 | 1.0-5.7 | |  |
|  |  |  |  |  |  |  |  |  |  |  |  | 0.08 |  |  |  |  |  |  | | **0.05** |
| **Mineral Wool Fibers ^a^** | | | | | | | | | | | | | | | | | | | | |
| Unexposed | 803 | 1167 | 1.0 |  |  |  | Unexposed | 803 | 1167 | 1.0 |  |  |  | Unexposed | 803 | 1167 | 1.0 |  |  | |
| Exposed | 19 | 12 | 2.5 | 1.1-5.4 | **0.02** |  | < 12.00 | 8 | 6 | 2.1 | 0.7-6.4 |  |  | < 0.06 | 7 | 5 | 2.1 | 0.7-7.1 |  | |
|  |  |  |  |  |  |  | > 12.00 | 11 | 6 | 2.9 | 1.0-8.2 |  |  | > 0.06 | 12 | 7 | 2.8 | 1.0-7.4 |  | |
|  |  |  |  |  |  |  |  |  |  |  |  | **0.02** |  |  |  |  |  |  | **0.02** | |
| **Brick Dust ^b^** | | | | | | | | | | | | | | | | | | | | |
| Unexposed | 753 | 1103 | 1.0 |  |  |  | Unexposed | 753 | 1103 | 1.0 |  |  |  | Unexposed | 753 | 1103 | 1.0 |  |  | |
| Exposed | 67 | 68 | 1.7 | 1.1-2.7 | **0.02** |  | < 11.00 | 26 | 37 | 1.3 | 0.7-2.3 |  |  | < 1.40 | 32 | 36 | 1.5 | 0.9-2.7 |  | |
|  |  |  |  |  |  |  | > 11.00 | 41 | 31 | 2.2 | 1.3-3.8 |  |  | > 1.40 | 35 | 32 | 2.0 | 1.1-3.5 |  | |
|  |  |  |  |  |  |  |  |  |  |  |  | **0.01** |  |  |  |  |  |  | **0.01** | |
| **Graphite Dust** | | | | | | | | | | | | | | | | | | | | |
| Unexposed | 813 | 1153 | 1.0 |  |  |  | Unexposed | 813 | 1153 | 1.0 |  |  |  | Unexposed | 813 | 1153 | 1.0 |  |  | |
| Exposed | 11 | 29 | 0.5 | 0.3-1.1 | 0.08 |  | < 11.00 | 9 | 10 | 1.2 | 0.5-3.1 |  |  | < 3.08 | 6 | 13 | 0.7 | 0.3-1.9 |  | |
|  |  |  |  |  |  |  | > 11.00 | 2 | 19 | 0.1 | 0.0-0.6 |  |  | > 3.08 | 5 | 16 | 0.4 | 0.1-1.1 |  | |
|  |  |  |  |  |  |  |  |  |  |  |  | **0.02** |  |  |  |  |  |  | 0.06 | |
| Abbreviations: BMI= body mass index; CI= confidence interval; N= number; OR= odds ratio. | | | | | | | | | | | | | | | | | | | | |
| Adjusted for age, sex, center, BMI, hypertension, and smoking status (ever, never). | | | | | | | | | | | | | | | | | | | | |
| Includes only high confidence exposures, those exposures assessed with a confidence of probable (40-90%) or definite (>90%). | | | | | | | | | | | | | | | | | | | | |
| Model also adjusted for occupational: ^a^asbestos exposure; ^b^concrete dust exposure. | | | | | | | | | | | | | | | | | | | | |
